# Predictive and prognostic value of preoperative serum tumor markers is EGFR mutation-specific in resectable non-small-cell lung cancer

**DOI:** 10.18632/oncotarget.8662

**Published:** 2016-04-09

**Authors:** Richeng Jiang, Xinyue Wang, Kai Li

**Affiliations:** ^1^ Tianjin Medical University Cancer Institute and Hospital, National Clinical Research Center for Cancer, Tianjin 300060, PR China; ^2^ Key Laboratory of Cancer Prevention and Therapy, Tianjin 300060, PR China; ^3^ Department of Thoracic Oncology, Tianjin Lung Cancer Center, Tianjin Cancer Institute & Hospital, Tianjin Medical University, Tianjin 300060, PR China

**Keywords:** CEA, Cyfra21-1, EGFR mutation, prognostic factor, non-small-cell lung cancer

## Abstract

**Background:**

The predictive and prognostic value of carcinoembryonic antigen (CEA), cytokeratin-19 fragments (Cyfra21-1), squamous cell carcinoma antigen (SCCA) and neuron-specific enolase (NSE) has been investigated in non-small-cell lung cancer (NSCLC) patients. However, few studies have directly focused on the association between these markers and epidermal growth factor receptor (EGFR) mutation status or mutation subtypes.

**Patients and methods:**

We retrospectively analyzed 1016 patients with stage I-IIIA NSCLC who underwent complete resection between 2008 and 2012. Correlations between serum tumor marker levels and EGFR mutations and survival parameters were analyzed and prognostic factors were identified.

**Results:**

Cyfra21-1 levels (*P* = 0.032 for disease-free survival [DFS]; *P* < 0.001 for overall survival [OS]) and clinical stage were identified as independent predictive and prognostic factors in EGFR-mutated adenocarcinoma patients. CEA levels (*P* < 0.001 for DFS; *P* = 0.002 for OS) and clinical stage were independently predictive and prognostic in EGFR wild-type adenocarcinoma patients. Further stratification analysis revealed that in EGFR exon 19 deletion adenocarcinomas, elevated Cyfra21-1 was an independent prognostic factor (*P* = 0.002). Within the Leu858Arg substitution subgroup, increased CEA (*P* = 0.005) and clinical stage were predictive factors of DFS, while elevated CEA (*P* = 0.005) and Cyfra21-1 (*P* = 0.027) were independent prognostic factors.

**Conclusion:**

Cyfra21-1 and CEA exhibit different predictive and prognostic values between EGFR-mutated and wild-type adenocarcinomas, as well as between EGFR mutation subtypes. The prognostic impact of preoperative serum tumor markers should be evaluated together with EGFR mutation status.

## INTRODUCTION

Despite significant progress in the last decade, lung cancer remains the leading cause of cancer-related death worldwide. Non-small cell lung cancer (NSCLC) is predominantly comprised of adenocarcinomas, squamous cell carcinomas and large cell lung carcinomas, and accounts for 75–85% of all lung cancers. Recent advancements in molecular targeted therapy have exploited the discovery of distinct cancer subsets with epidermal growth factor receptor (EGFR) mutations and led to a major paradigm shift in the treatment of NSCLC [1, 2]. EGFR exon 19 deletion (del19) and exon 21 Leu858Arg substitution (L858R) make up around 90% of all EGFR mutation-positive lung adenocarcinomas, and are strongly associated with robust responses and improved progression-free survival (PFS) to EGFR tyrosine kinase inhibitors (EGFR-TKIs) [3]. Moreover, several studies demonstrated that EGFR del19 mutation consistently associated with better EGFR-TKI therapeutic outcomes compared with L858R substitution [4–6]. Preclinical studies have shown that EGFR del19 and L858R mutants have distinct biochemical properties that affect response to EGFR-TKIs [7], thus likely belong to different molecular subsets and should be studied independently [4].

EGFR mutations are most common in Asian populations, nonsmokers, females and those with adenocarcinoma histology [1]. The EGFR mutation rate in squamous cell carcinoma is reported to be approximately 5%, therefore, most squamous cell carcinoma patients do not benefit from EGFR targeted therapy [8]. Moreover, even in squamous cell carcinoma patients who achieved a partial response by EGFR-TKI, the median PFS was shorter than patients with adenocarcinoma harboring EGFR mutations [9].

Serum tumor markers including carcinoembryonic antigen (CEA) [10, 11], neuron-specific enolase (NSE) [12], cytokeratin-19 fragments (Cyfra21-1) [13, 14] and squamous cell carcinoma antigen (SCCA) [15], have been considered to be predictive or prognostic in NSCLC, although no consensus has been reached. Several studies have shown that serum tumor markers were associated with EGFR mutation status and capable of predicting the efficacy of EGFR-TKI therapy in advanced NSCLC. Higher serum CEA level has been associated with higher EGFR mutation rate [16–18], higher disease control rate (DCR) and longer survival time in advanced adenocarcinoma patients treated with EGFR-TKI [19]. Furthermore, in advanced NSCLC patients treated with EGFR-TKIs, high CEA and/or low Cyfra21-1 levels significantly correlated with higher responses and longer survival, especially in patients with unknown EGFR mutation status or with squamous cell carcinoma diagnosis [20]. Similarly, CEA reduction after 1 month of EGFR-TKI therapy was significantly correlated with DCR and PFS in EGFR wild-type/unknown but not in mutated NSCLC cases [21]. However, Fiala et al. reported that high pre-treatment levels of CEA and/or Cyfra21-1 were associated with poor outcome for advanced NSCLC patients treated with erlotinib [22]. Moreover, Tanaka et al. demonstrated that high pretreatment Cyfra21-1, but not CEA levels, closely associated with shorter PFS in EGFR-TKI treated EGFR-mutated NSCLC patients [8]. Therefore, prognostic significance of CEA and Cyfra21-1 in EGFR mutated NSCLC remains largely unknown. Furthermore, the relationship between NSE, SCCA and EGFR mutation has not been well investigated.

We investigated indicative prognostic factors among serum tumor markers (CEA, NSE, SCCA and Cyfra21-1) and the clinical characteristics for NSCLC patients who underwent complete surgical resection of stage I, II and IIIA tumors. Particularly we addressed the impact of respective tumor markers in patients with EGFR mutations as potential prognostic factors.

## RESULTS

### Patient characteristics

The initial study cohort included 1016 consecutive patients with completely resected NSCLC, and the follow-up period ranged from 2 to 72 months. Clinicopathological characteristics of the patients are shown in Table 1. Included were 616 males and 400 females, with a mean age of 59.3 years (range: 20–89 years). Among the patient histology, 566 (55.7%) were adenocarcinomas, 352 (34.6%) squamous cell carcinomas, 42 (4.2%) large cell lung carcinomas, 19 (1.9%) adenosquamous carcinomas, and 37 (3.6%) other carcinoma types. The distribution of clinical stages was as follows: 508 stage I, 198 stage II, and 310 stage IIIA. Eight hundred fifteen patients received lobectomy, 143 underwent pneumonectomy, and 58 wedge resection. EGFR Mutations were detected in 38.5% (218/566) adenocarcinoma cases, 4.3% (15/352) squamous cell carcinoma, 42.1% (8/19) adenosquamous carcinoma and 9.5% (4/42) large cell lung carcinoma. Among the 245 EGFR mutations, 123 were del19, and 122 were L858R.

**Table 1:** Characteristics and clinicopathological data for the 1016 NSCLC patients

Variable	Patients	%
**Gender**		
Female	400	39.4
Male	616	60.6
**Age (years)**		
≤ 60	518	51
> 60	498	49
**Smoking history**		
Never	430	42.3
Former/current	586	57.7
**Clinical Stage**		
I	508	50
II	198	19.5
IIIA	310	30.5
**Tumor size**		
≤ 3 cm	500	49.2
> 3 cm	516	50.8
**Regional lymph node metastasis**		
No	631	62.1
Yes	385	37.9
**Histology**		
adenocarcinoma	566	55.7
squamous cell carcinoma	352	34.6
large cell lung carcinoma	42	4.2
adenosquamous carcinoma	19	1.9
Others	37	3.6
**EGFR mutation**		
Exon 19 deletion	123	12.1
L858R substitution	122	12
Wild-type	771	75.9
**Surgical Resection**		
Pneumonectomy	143	14.1
Lobectomy	815	80.2
Wedge resection	58	5.7
**Adjuvant treatment**		
Chemotherapy	699	68.8
Postoperative radiotherapy	15	1.5
Chemoradiotherapy	140	13.8
Others/none	162	15.9
**CEA**		
≤ 5.0 ng/ml	676	66.5
> 5.0 ng/ml	340	33.5
Cyfra21-1		
≤ 3.3 ng/ml	572	56.3
> 3.3 ng/ml	444	43.7
**SCCA**		
≤ 1.5 ng/ml	834	82.1
> 1.5 ng/ml	182	17.9
**NSE**		
≤ 15.2 ng/ml	665	65.5
> 15.2 ng/ml	351	34.5
**Total**	1016	100

Of the 1016 NSCLC patients, 699 (68.8%) received platinum-based adjuvant chemotherapy, 15 (1.5%) received postoperative radiotherapy, 140 (13.8%) received platinum-based adjuvant chemoradiotherapy, and 549 (54.0%) had recurrent disease during the study follow-up period. Median DFS was 35.0 months. Four hundred and seventy six (86.7%) recurrent patients whose condition permitted receiving systemic chemotherapy, 76 (13.8%) patients also received radiotherapy as local therapy, 54 (9.8%) received EGFR-TKIs and one patient received anaplastic lymphoma kinase inhibitor. At the end of the last follow up, 454 patients had died. Median overall survival was 52.7 months, and the 5-year survival rate was 45.2% for the whole study population.

### Clinicopathological characteristics and serum tumor marker levels

Among all patients, preoperative CEA, Cyfra21-1, SCCA, and NSE levels were elevated in 33.5%, 43.7%, 17.9% and 34.5% of patients, respectively. These rates differed between adenocarcinoma and squamous cell carcinoma histological types. The positive rate of CEA was significantly lower in squamous cell carcinomas than in adenocarcinomas (27.0% versus 58.5%, χ^2^ = 3.981, *P* < 0.001), while the positive rates of increased Cyfra21-1, SCCA as well as NSE were significantly higher in squamous cell carcinomas compared to adenocarcinomas (*P* < 0.001 for all comparisons). According to clinical stage, patients with stages II and IIIA tended to have higher tumor marker values than those with stage I. The distributions of all serum markers tested according to tumor stage and histology are shown in Table 2.

**Table 2:** The distributions of serum tumor markers according to tumor stage, histology and EGFR mutation

	CEA Median (range)	CEA > 5 ng/ml (%)	CFRA 21-1 Median (range)	CYFRA21-1> 3.3 ng/ml (%)	SCCA Median (range)	SCCA > 1.5 ng/ml (%)	NSE Median (range)	NSE > 15.2 ng/ml (%)
**All patients (*n* = 1016)Histology**								
Adenocarcinoma	3.42 (0.33–959.6)	209 (36.9)	2.65 (0.30–138.2)	181 (32.0)	0.70 (0.10–29.53)	41 (7.2)	13.73 (1.03–112.04)	155 (27.4)
Squamous cell carcinoma	3.20 (0.20–499.8)	95 (27.0)	4.18 (0.30–936.0)	228 (64.8)	1.10 (0.10–58.40)	126 (35.8)	14.81 (0.90–59.72)	153 (43.5)
Large cell lung carcinoma	3.74 (0.66–52.20)	16 (38.1)	3.09 (1.02–10.26)	16 (38.1)	1.00 (0.40–14.50)	9 (21.4)	14.54 (8.96–28.01)	16 (38.1)
Adenosquamous carcinoma	6.24 (1.37–260.8)	12 (63.2)	0.80 (0.10–58.40)	5 (13.5)	0.80 (0.10–10.30)	3 (15.8)	16.21 (12.76–29.91)	12 (63.2)
Others	2.26 (0.56–14.96)	8 (21.6)	2.28 (0.46–61.33)	14 (73.7)	0.80 (0.10–40.94)	3 (8.1)	14.26 (6.41–26.92)	15 (40.5)
**Clinical stage**								
I	2.93 (0.20–283.3)	129 (25.4)	2.75 (0.30–936.0)	172 (33.9)	0.80 (0.10–58.40)	63 (12.4)	13.67 (0.90–112.04)	140 (27.6)
II	3.54 (0.60–389.5)	71 (35.9)	3.93 (0.30–71.59)	120 (60.6)	0.90 (0.10–54.70)	61 (30.8)	14.68 (1.77–67.90)	85 (42.9)
IIIA	4.43 (0.42–959.6)	140 (45.2)	3.26 (0.46–138.2)	152 (49.0)	0.80 (0.10–32.00)	58 (18.7)	14.54 (1.03–86.17)	126 (40.6)
Total	3.30 (0.20–959.6)		3.06 (0.30–936.0)		0.80 (0.10–58.40)		14.24 (0.90–112.04)	
**AD patients (*n* = 566)Mutation status**								
EGFR-mutated	3.48 (0.33–959.6)	86 (39.4)	2.50 (0.74–138.2)	68 (31.2)	0.60 (0.10–22.75)	12 (5.5)	14.27 (1.77–112.04)	70 (32.1)
EGFR wild-type	3.40 (0.42–389.5)	123 (35.3)	2.68 (0.30–43.32)	113 (32.5)	0.70 (0.10–29.53)	29 (8.3)	13.25 (1.03–86.17)	85 (24.4)
Total	3.42 (0.33–959.6)	209 (36.9)	2.65 (0.30–138.2)	181 (32.0)	0.70 (0.10–29.53)	41 (7.2)	13.73 (1.03–112.04)	155 (27.4)

Median levels and positive rates for CEA, Cyfra21-1 or SCCA were similar regardless of EGFR mutation status in adenocarcinoma patients (Table 2). Similarly, no differences were found in those marker levels between del19 and L858R adenocarcinoma patient subgroups. However, median NSE levels in patients with EGFR mutations were higher than those with wild-type EGFR (median: 14.27 versus 13.25 ng/ml, *P* = 0.007). In addition, elevated NSE was observed in 32.1% EGFR mutated patients compared to 24.4% in EGFR wild-type patients (χ^2^ = 3.981, *P* = 0.046). No difference was found in median levels and positive rates of NSE between del19 and L858R adenocarcinoma subgroups.

### Association of CEA/Cyfra21-1/NSE with DFS and OS in adenocarcinoma or squamous cell carcinoma patients

Among the 566 adenocarcinoma patients, 209 had elevated CEA levels, 181 elevated Cyfra21-1, 41 elevated SCCA, and 155 elevated NSE. Median DFS as well as OS were significantly shorter in patients with elevated CEA (18.1 versus 51.8 months, log-rank χ^2^ = 59.948, *P* < 0.001 for DFS; 39.6 months versus Not Reached [NR], log-rank χ^2^ = 37.065, *P* < 0.001 for OS). A similar inverse relationship with DFS and OS was observed for Cyfra21-1 (24.0 versus 44.6 months, log-rank χ^2^ = 34.852, *P* < 0.001 for DFS; 39.6 months versus NR, log-rank χ^2^ = 30.169, *P* < 0.001 for OS). Patients with high SCCA had significantly shorter DFS (22.0 versus 36.0 months, log-rank χ^2^ = 4.542, *P* = 0.033), but this was not associated with an effect on OS (53.8 versus 59.8 months, log-rank χ^2^ = 1.665, *P* = 0.197). Elevated NSE patients did not exhibit any difference in DFS (*P* = 0.473) nor OS (*P* = 0.268) compared to those with normal levels.

Of the 352 squamous cell carcinoma patients, increased levels of CEA, Cyfra21-1, SCCA, and NSE were observed in 95, 228, 126, and 153 patients, respectively. Elevated SCCA levels were significantly associated with shorter DFS and OS in these patients (17.2 versus 57.8 months, log-rank χ^2^ = 11.537, *P* = 0.001 for DFS; 35.6 versus 61.9 months, log-rank χ^2^ = 9.622, *P* = 0.002 for OS), while increased CEA correlated with worse DFS (24.0 versus 46.0 months, log-rank χ^2^ = 4.411, *P* = 0.036) but not OS (*P* = 0.056). Neither Cyfra21-1 nor NSE positivity was correlated with any effect on DFS or OS.

### Association of increased CEA/Cyfra21-1/NSE with DFS and OS based on EGFR mutation status

EGFR was mutated in 218 adenocarcinoma patients, and among them CEA, Cyfra21-1, SCCA, and NSE increased in 86, 68, 12, and 70 patients, respectively. Due to the low frequency of elevated SCCA in EGFR-mutated NSCLC, we thereafter only investigated whether other markers could predict clinical outcome.

**Figure 1:**
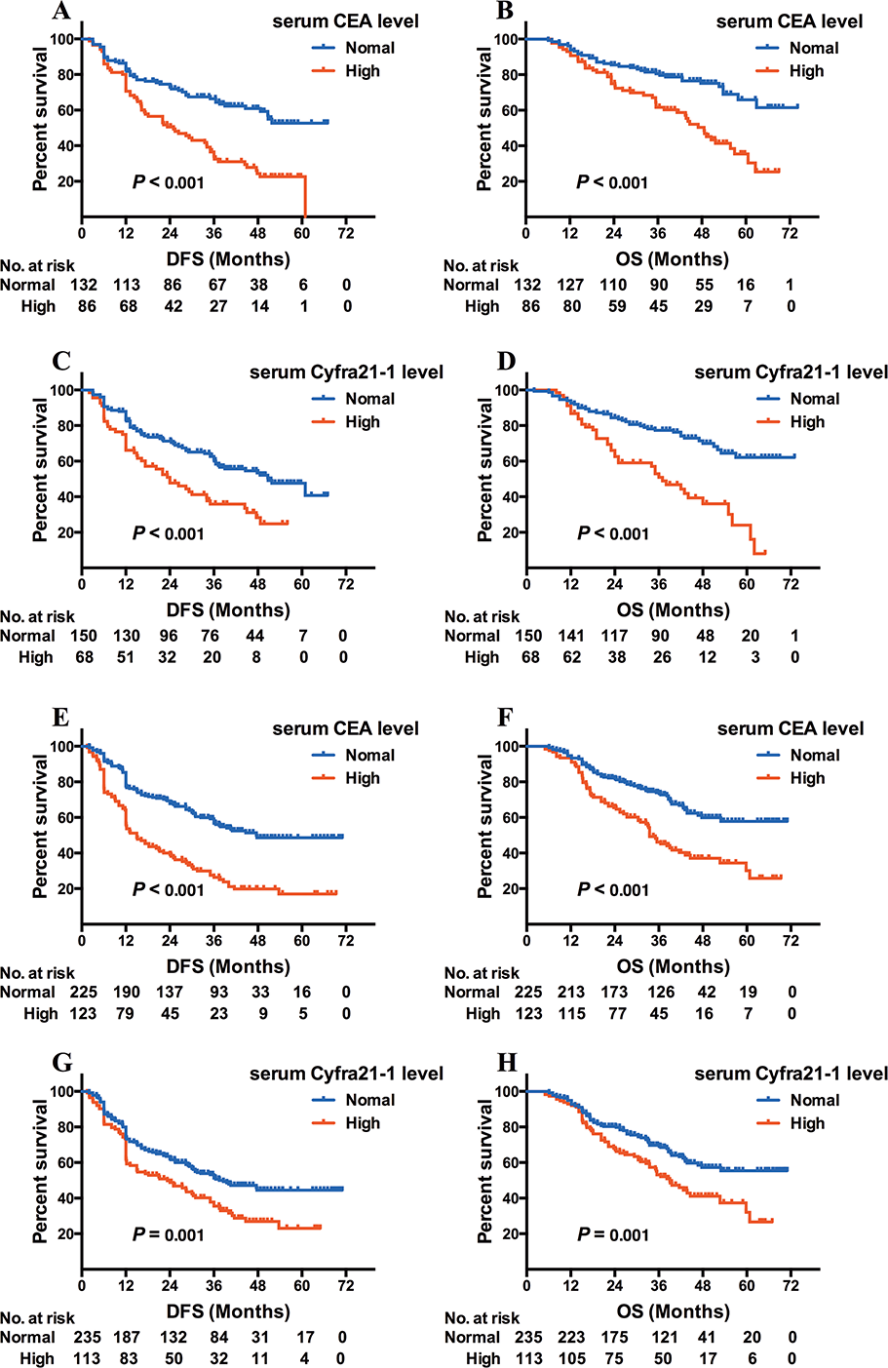
Kaplan-Meier survival curves of DFS and OS based on CEA/Cyfra21-1 levels in EGFR-mutated or wild-type adenocarcinoma patients DFS (**A**) and OS (**B**) based on CEA level in adenocarcinoma patients with EGFR mutation; DFS (**C**) and OS (**D**) based on Cyfra21-1 level in adenocarcinoma patients with EGFR mutation; DFS (**E**) and OS (**F**) based on CEA level in EGFR wild-type adenocarcinoma patients; DFS (**G**) and OS (**H**) based Cyfra21-1 level in EGFR wild-type adenocarcinoma patients.

EGFR-mutated adenocarcinoma patients with either elevated CEA or Cyfra21-1 exhibited both shorter DFS and OS (CEA: 25.0 versus 46.4 months, log-rank χ^2^ = 21.977, *P* < 0.001 for DFS, Figure 1A; 48.6 months versus NR, log-rank χ^2^ = 16.315, *P* < 0.001 for OS, Figure 1B; Cyfra21-1: 24.0 versus 50.8 months, log-rank χ^2^ = 12.820, *P* < 0.001 for DFS, Figure 1C; 42.6 months versus NR, log-rank χ^2^ = 23.537, *P* < 0.001 for OS, Figure 1D). Abnormal NSE levels were not associated with DFS or OS for this patient population.

The remaining 348 adenocarcinoma patients were EGFR wild-type and CEA, Cyfra21-1, and NSE elevated in 123, 113, and 85 patients, respectively. Similar to EGFR-mutated patients, both CEA and Cyfra21-1 increases were associated with worse DFS and OS (CEA: 15.0 versus 47.7 months, log-rank χ^2^ = 40.887, *P* < 0.001 for DFS; Figure 1E; 33.5 versus 56.3 months, log-rank χ^2^ = 22.726, *P* < 0.001 for OS, Figure 1F; Cyfra21-1: 23.3 versus 37.5 months, log-rank χ^2^ = 10.155, *P* = 0.001 for DFS, Figure 1G; 39.0 versus 54.7 months, log-rank χ^2^ = 10.399, *P* = 0.001 for OS, Figure 1H). No relationship was found between increased NSE and DFS or OS.

### Association of elevated CEA/Cyfra21-1/NSE with DFS and OS in EGFR-mutant subtypes

**Figure 2:**
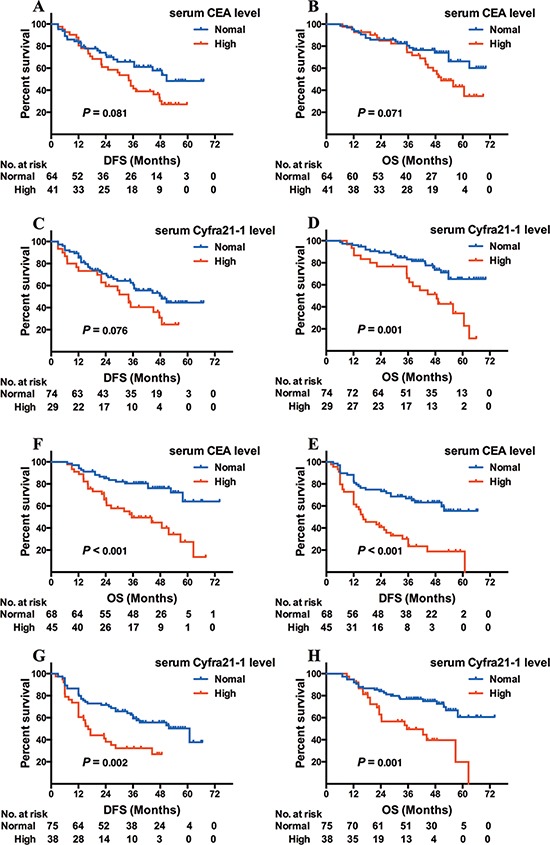
Kaplan-Meier survival curves of DFS and OS based on CEA/Cyfra21–1 levels in EGFR del19 or L858R adenocarcinoma patients DFS (**A**) and OS (**B**) based on CEA level in del19 adenocarcinoma patients; DFS (**C**) and OS (**D**) based on Cyfra21–1 level in del19 adenocarcinoma patients; DFS (**E**) and OS (**F**) based on CEA level in L858R adenocarcinoma patients; DFS (**G**) and OS (**H**) based on Cyfra21–1 level in L858R adenocarcinoma patients.

Among the 218 patients with EGFR mutations, 105 patients possessed the del19 mutation. Of these cases, 64 had increased CEA, 75 increased Cyfra21-1, and 37 increased NSE. Interestingly, there was no association in DFS with elevated levels of any of the 3 markers (*P* = 0.081 for CEA, Figure 2A; *P* = 0.076 for Cyfra21-1, Figure 2C; *P* = 0.849 for NSE). In addition, there was no effect on OS for increased CEA (*P* = 0.071; Figure 2B) or NSE (*P* = 0.958) patients, but elevated Cyfra21-1 was associated with shorter OS (48.6 months versus NR, log-rank χ^2^ = 10.267, *P* = 0.001; Figure 2D).

The remaining EGFR-mutated patients harbored L858R, of which 45 had increased CEA, 38 increased Cyfra21-1 and 33 increased NSE. Both CEA and Cyfra21-1 increases were associated with shorter DFS and OS (CEA: 16.3 versus 52.9 months, log-rank χ^2^ = 19.376, *P* < 0.001 for DFS, Figure 2E; 35.5 versus NR, log-rank χ^2^ = 14.625, *P* < 0.001 for OS, Figure 2F; Cyfra21-1: 16.3 versus 60.9 months, log-rank χ^2^ = 9.284 *P* = 0.002 for DFS, Figure 2G; 35.5 versus 63.4 months, log-rank χ^2^ = 11.818, *P* = 0.001 for OS, Figure 2H). Elevated NSE was not linked to worse DFS nor OS (*P* = 0.061 for DFS; *P* = 0.070 for OS).

### Univariate and multivariate analysis of prognostic factors in adenocarcinoma patients

By univariate analysis, adenocarcinoma patients harboring EGFR mutations had DFS and OS significantly associated with clinical stage, tumor size, regional lymph node (LN) metastasis, adjuvant treatment, CEA, and Cyfra21-1 (Table 3). When stratifying EGFR-mutated cases into del19 (105 cases) and L858R (113 cases) subgroups, we found that DFS and OS were longer in the Cyfra21-1 normal group than in the elevated Cyfra21-1 group for both subgroups. However, elevated CEA level was associated with shorter DFS and OS only in L858R patients. In addition, we found that DFS and OS significantly associated with clinical stage, tumor size, regional LN metastasis, adjuvant treatment, CEA, and Cyfra21-1 in the EGFR wild-type adenocarcinoma patients (Table 3).

**Table 3:** Univariate analysis of DFS and OS for 218 EGFR mutated and 348 EGFR wild-type adenocarcinoma patients

Variable	EGFR mutation					EGFR wild-type				
	*n*	Median DFS (mo)	*P*-value	Median OS (mo)	*P*-value	*n*	Median DFS (mo)	*P*-value	Median OS (mo)	*P*-value
**Total**	**218**					**348**				
**Gender**										
Female	138	45	0.143	63.6	0.218	164	30.4	0.908	52.9	0.812
Male	80	35		56.9		184	36		52.9	
**Age (years)**										
≤ 60	132	36.5	0.356	60.6	0.18	178	30	0.849	52.7	0.979
> 60	86	47.8		66.7		170	36		59.8	
**Smoking history**										
Never	129	36.5	0.437	65.6	0.751	159	32.4	0.633	52.7	0.637
Former/current	89	44.4		56.9		189	31		52.9	
**Clinical Stage**										
I	120	46.5	**< 0.001**	NR	**< 0.001**	177	46.5	**< 0.001**	NR	**< 0.001**
II	30	37.2		53.8		54	30.4		59.8	
IIIa	68	23.3		43.7		117	15.2		38.2	
**Tumor size**										
≤ 3 cm	135	47.6	0.067	64.6	**0.028**	211	36	0.22	59.8	0.774
> 3 cm	83	30		49.2		137	30.4		44.6	
**Regional lymph node metastasis**										
No	134	50.8	**< 0.001**	66.1	**< 0.001**	207	44.9	**< 0.001**	56	**0.001**
Yes	84	26.4		48.6		141	23		39.5	
**EGFR mutation subtype**										
Exon 19 deletion	105	44.6	0.704	62.9	0.362					
L858R substitution	113	37.6		57.9						
**Surgical Resection**										
Pneumonectomy	6	12	0.101	35.5	0.216	36	21	0.315	33.5	0.426
Lobectomy	184	47.6		62.9		296	36		59.8	
Wedge resection	28	36		51.7		16	24.3		39.5	
**Adjuvant treatment**										
Chemotherapy	120	44.4	**< 0.001**	60.6	**0.001**	236	35	**< 0.001**	59.8	**< 0.001**
Postoperative radiotherapy	6	12.2		32.5		2	11.2		11.2	
Chemoradiotherapy	29	21.3		43.5		61	12.2		33.5	
Others/none	63	50.8		66.5		49	49		58.4	
**CEA**										
≤ 5.0 ng/ml	132	46.4	**< 0.001**	NR	**< 0.001**	225	47.7	**< 0.001**	NR	**< 0.001**
> 5.0 ng/ml	86	25		48.6		123	15		33.5	
**Cyfra21–1**										
≤ 3.3 ng/ml	150	50.8	**< 0.001**	NR	**< 0.001**	235	37.5	**0.001**	54.7	**0.001**
> 3.3 ng/ml	68	24		42.6		113	23.3		39	
**SCCA**										
≤ 1.5 ng/ml	206	44.4	0.102	62.6	0.147	319	35	0.162	52.9	0.646
> 1.5 ng/ml	12	21.3		35.4		29	30.4		53.3	
**NSE**										
≤ 15.2 ng/ml	148	48.5	0.264	NR	0.216	263	30.9	0.833	52.9	0.425
> 15.2 ng/ml	70	35		55.8		85	36		39.5	
**Exon 19 deletion subgroup (*n* = 105)CEA**										
≤ 5.0 ng/ml	64	50.7	0.081	NR	0.071					
> 5.0 ng/ml	41	34.1		50.5						
**Cyfra21–1**										
≤ 3.3 ng/ml	75	48.6	0.076	NR	**0.001**					
> 3.3 ng/ml	30	34		48.6						
**NSE**										
≤ 15.2 ng/ml	68	37.2	0.849	59	0.958					
> 15.2 ng/ml	37	45		60.6						
**L858R substitution subgroup (*n* = 113)CEA**										
≤ 5.0 ng/ml	68	52.9	**< 0.001**	NR	**< 0.001**					
> 5.0 ng/ml	45	16.3		35.5						
**Cyfra21–1**										
≤ 3.3 ng/ml	75	60.9	**0.002**	63.4	**0.001**					
> 3.3 ng/ml	38	16.3		35.5						
**NSE**										
≤ 15.2 ng/ml	80	51.8	0.061	57.9	0.07					
> 15.2 ng/ml	33	26.4		51.7						

By multivariate analysis, Cyfra21-1 (HR = 1.543, *P* = 0.032 for DFS; HR = 2.527, *P* < 0.001 for OS) and clinical stage (HR = 1.825, *P* = 0.004 for DFS; HR = 1.694, *P* = 0.040 for OS) were independent predictive and prognostic factors in all EGFR-mutated adenocarcinoma patients. Further stratification analysis revealed that Cyfra21-1 (HR = 2.713, *P* = 0.002) was an independent prognostic factor for EGFR del19 adenocarcinoma patients. In L858R subgroup, CEA (HR = 2.259, *P* = 0.005) and clinical stage (HR = 2.234, *P* = 0.007) were predictive factors of DFS, while CEA (HR = 2.515, *P* = 0.005) and Cyfra21-1 (HR = 2.068, *P* = 0.027) were independent prognostic factors for OS (Table 4). For EGFR wild-type adenocarcinoma patients, CEA (HR = 2.150, *P* < 0.001 for DFS; HR = 1.711, *P* = 0.002 for OS) and clinical stage (HR = 1.527, *P* = 0.020 for DFS; HR = 1.593, *P* = 0.026 for OS) were independently predictive and prognostic.

**Table 4:** Multivariate analysis of DFS and OS for adenocacinoma patients harboring EGFR mutations

Variable	DFS		OS	
	HR (95% CI)	*P*-value	HR (95% CI)	*P*-value
**EGFR-mutated (*n* = 218)**				
Clinical Stage (I–II vs. IIIA)	1.825 (1.205–2.762)	0.004	1.694 (1.025–2.802)	0.040
CEA (> 5 ng/ml vs. ≤ 5 ng/ml)				
Cyfra21–1 (> 3.3 ng/ml vs.≤ 3.3 ng/ml)	1.543 (1.039–2.292)	0.032	2.527 (1.620–3.942)	< 0.001
**Exon 19 deletion subgroup (*n* = 105)**				
Clinical Stage (I–II vs. IIIA)				
CEA (> 5 ng/ml vs. ≤ 5 ng/ml)				
Cyfra21–1 (> 3.3 ng/ml vs.≤ 3.3 ng/ml)			2.713 (1.229–4.428)	0.002
**L858R substitution subgroup (*n* = 113)**				
Clinical Stage (I–II vs. IIIA)	2.234 (1.246–4.004)	0.007		
CEA (> 5 ng/ml vs. ≤ 5 ng/ml)	2.259 (1.277–3.996)	0.005	2.515 (1.313–4.819)	0.005
Cyfra21–1 (> 3.3 ng/ml vs.≤ 3.3 ng/ml)			2.068 (1.087–3.933)	0.027
**EGFR wild-type (*n* = 348)**				
Clinical Stage (I–II vs. IIIA)	1.527 (1.070–2.181)	0.02	1.593 (1.058–2.398)	0.026
CEA (> 5 ng/ml vs. ≤ 5 ng/ml)	2.150 (1.606–2.878)	< 0.001	1.711 (1.224–2.392)	0.002
Cyfra21–1 (> 3.3 ng/ml vs. ≤ 3.3 ng/ml)				

### Patients treated with EGFR-TKI therapy

Over 25% (139/549) of recurrent patients harbored EGFR mutations, 54 of which received EGFR-TKI therapy (30 del19 and 24 L858R cases), either gefitinib 250 mg/d or erlotinib 150 mg/d. Eighteen patients were treated with EGFR-TKI as a first-line therapy, 27 as a second-line therapy, and 9 as third-line or thereafter. No significant difference was observed in post-recurrence survival (PRS) between recurrent EGFR-mutated patients with and without EGFR-TKI therapy (median PRS, 15.1 versus 10.4 months, log-rank χ^2^ = 2.413, *P* = 0.120). It is noteworthy that serum CEA or NSE could not predict PRS for patients treated with EGFR-TKIs, while elevated Cyfra21-1 conferred shorter PRS (log-rank χ^2^ = 8.110, *P* = 0.004).

### Non-adenocarcinoma patients harboring EGFR mutations

Among non-adenocarcinoma patients, EGFR mutations were detected in 15 squamous cell carcinomas, 8 adenosquamous carcinomas 8 and 4 large cell lung carcinomas (Table 5). Non-adenocarcinoma patients harboring EGFR mutations had significantly shorter DFS and OS compared to EGFR-mutated adenocarcinoma patients (15.0 versus 39.0 months, log-rank χ^2^ = 15.075, *P* < 0.001 for DFS; 30.1 versus 62.6 months, log-rank χ^2^ = 32.665, *P* < 0.001 for OS). In addition, EGFR-mutated squamous cell carcinoma patients had significantly worse DFS and OS compared to EGFR wild-type squamous cell carcinoma patients (15.0 versus 44.6 months, log-rank χ^2^ =3.839, *P* = 0.050 for DFS; 30.1 versus 52.9 months, log-rank χ^2^ = 7.870, *P* = 0.005 for OS).

**Table 5:** Clinical parameters and survival of non-adenocarcinoma patients harboring EGFR mutations

Histology	Mutation status	Age (yr)/Sex (M, W)	Clinical Stage	Cyfra21–1 (ng/ml)	CEA (ng/ml)	SCCA (ng/ml)	NSE (ng/ml)	DFS (mo)	OS (mo)
Squamous cell carcinoma	Exon 19 deletion	55/W	I	1.69	30.74	0.3	7.25	24	43.7
Squamous cell carcinoma	Exon 19 deletion	55/M	II	39.38	2.32	0.6	33.36	9.1	16.2
Squamous cell carcinoma	Exon 19 deletion	55/W	I	3.2	4.29	0.6	19.64	65	65
Squamous cell carcinoma	Exon 19 deletion	55/W	IIIA	14.55	16.94	0.7	29.81	36.1	37.6
Squamous cell carcinoma	Exon 19 deletion	52/W	IIIA	21.27	17.08	18.6	12.81	23.9	32.7
Squamous cell carcinoma	Exon 19 deletion	68/M	I	8.04	4.52	0.8	17.08	15	18.3
Squamous cell carcinoma	Exon 19 deletion	69/W	I	3.86	4.05	0.4	10.3	14	24.4
Squamous cell carcinoma	Exon 19 deletion	71/M	IIIA	6.28	2.12	1.6	8.38	13.2	33.5
Squamous cell carcinoma	Exon 19 deletion	67/W	IIIA	14.74	2.08	5.5	13.47	12	13.2
Squamous cell carcinoma	Exon 19 deletion	52/W	I	0.4	232.89	0.8	15.33	22	30.1
Squamous cell carcinoma	Exon 19 deletion	57/M	IIIA	3.12	2.66	0.6	14.24	12	17.3
Squamous cell carcinoma	L858R substitution	48/W	IIIA	7.7	13.44	0.6	33.95	5	7.1
Squamous cell carcinoma	L858R substitution	55/W	I	9.91	255.4	0.3	33.49	2	16.2
Squamous cell carcinoma	L858R substitution	56/W	I	2.64	31.4	0.8	12.3	28.3	28.3
Squamous cell carcinoma	L858R substitution	69/M	II	2.38	2.39	1.3	10.67	21.2	21.2
Adenosquamous carcinoma	Exon 19 deletion	54/W	IIIA	15.68	10.83	0.3	29.91	45.2	50.7
Adenosquamous carcinoma	Exon 19 deletion	45/W	IIIA	3.89	69.86	0.6	14.52	54.7	54.7
Adenosquamous carcinoma	Exon 19 deletion	51/W	IIIA	2.14	4.35	0.4	15.35	12	32.5
Adenosquamous carcinoma	Exon 19 deletion	58/W	II	2.47	5.23	0.7	17.94	21.3	39.6
Adenosquamous carcinoma	Exon 19 deletion	48/W	IIIA	1.84	16.04	0.5	12.76	37.5	43.7
Adenosquamous carcinoma	Exon 19 deletion	64/M	II	3.91	6.24	0.5	21.18	12	23.4
Adenosquamous carcinoma	L858R substitution	61/M	II	14.95	14.87	1	14.97	35	38.6
Adenosquamous carcinoma	L858R substitution	63/W	IIIA	5.95	26.41	0.1	15.74	25	23.4
Large cell lung carcinoma	Exon 19 deletion	34/W	IIIA	1.41	6.17	0.4	18.16	3	12.2
Large cell lung carcinoma	L858R substitution	65/W	II	4.08	1.72	0.8	12.49	5	20.3
Large cell lung carcinoma	L858R substitution	71/W	I	10.26	42.38	0.8	25.28	3	3
Large cell lung carcinoma	L858R substitution	72/M	I	3.6	2.22	1.2	17.89	3	9.1

Serum Cyfra21-1 in 27 non-adenocarcinoma EGFR-mutated patients was significantly higher than in 218 EGFR-mutated adenocarcinoma cases (median: 3.91 versus 2.50 ng/ml, *P* = 0.014). Among the non-adenocarcinoma patients, those with elevated Cyfra21-1 had shorter DFS and OS compared to normal Cyfra21-1 cases (median DFS, 13.2 versus 22.0 months; median OS, 23.4 versus 39.6 months), especially among the 15 squamous cell carcinoma patients, 9 cases with elevated Cyfra21-1 had shorter DFS and OS compared to those with normal Cyfra21-1 levels (median DFS, 13.2 versus 24.0 months; median OS, 18.3 versus 43.7 months). Serum SCCA levels were not different between squamous cell carcinoma patients with or without EGFR mutations, nor did it have a relationship with DFS or OS.

## DISCUSSION

NSCLC is a heterogeneous and complex disease with high genetic, epigenetic, and phenotypic diversity. Preoperative serum tumor markers might reflect substantial intratumor subclonal variability, and have been evaluated as predictive or prognostic factors, either alone or in combination with clinicopathological parameters. Moreover, several serum tumor markers have been shown to be associated with EGFR mutation status and efficacy of EGFR-TKI treatment. However, there is not a consensus regarding the predictive and prognostic significance of these markers. In addition, their association with EGFR mutation subtypes (del19 and L858R) remains largely unknown.

In this study, we demonstrated that elevated Cyfra21-1 and advanced clinical stage were independently associated with shorter DFS and OS in EGFR-mutated adenocarcinoma patients, while increased CEA and advanced clinical stage were independently associated with worse DFS and OS in wild-type adenocarcinoma patients. Within the EGFR del19 subgroup, elevated Cyfra21-1 was correlated with shorter OS. However in L858R group, CEA and clinical stage were significantly associated with DFS, while elevated CEA and Cyfra21-1 were significantly unfavorable prognostic factors.

The correlation between baseline tumor marker values and NSCLC survival is controversial [10–13, 15, 21, 23–26]. We observed a direct relationship between high CEA and unfavorable prognosis, but only for adenocarcinoma patients with wild-type EGFR. Preoperative CEA may not be predictive or prognostic for patients with EGFR mutations due to the high sensitivity to EGFR-TKI and platinum-based doublet chemotherapy [27, 28].

NSCLC diagnoses are based on biopsies from small regions of the tumor, thus may underrepresent rare subclonal squamous cell carcinoma or neuroendocrine cell populations. Simultaneous measurement of several serum tumor markers might provide a more comprehensive measure of a tumor's biological potential. Cyfra21-1 was previously shown to be useful for predicting clinical outcome of NSCLC patients [25], especially those with EGFR mutations [8]. Consistently, we observed that only in EGFR-mutated adenocarcinoma patients, elevated Cyfra21-1 was associated with worse DFS and OS. It is tempting to speculate that histologic heterogeneity plays a critical role in the elevated treatment efficacy for these patients compared to wild-type EGFR patients where Cyfra21-1 is not prognostic.

We observed squamous cell carcinoma patients had significantly shorter DFS and OS than those with adenocarcinomas, especially in the context of EGFR mutations, consistent with previous reports [8, 25]. Elevated Cyfra21-1 reliably predicted worse DFS and OS in EGFR-mutated squamous cell carcinoma patients.

Previous studies have linked EGFR mutation status to elevated CEA levels [29, 30]. However, we were unable to found a difference in the CEA or Cyfra21-1 levels between EGFR-mutated or wild-type adenocarcinoma patients [20]. This discrepancy could be attributed to our large sample size that partly avoided possible selection bias.

To our knowledge, the association between serum tumor markers and EGFR mutation subtypes (del19 and L858R) has not been reported. Previous studies have demonstrated that unlike the L858R substitution, EGFR del19 mutation is consistently associated with improved EGFR-TKI therapeutic outcomes [4–6]. This suggests that EGFR del19 and L858R NSCLC possess different biochemical properties and belong to distinct molecular subsets. Our findings are consistent with this notion as only Cyfra21-1 in correlated with shorter OS in the EGFR del19 group while both CEA and Cyfra21-1 were prognostic in L858R group. Even when using a more stringent cut-off (20.0 ng/ml), elevated CEA serum levels were still not significantly associated with either DFS (*P* = 0.149) nor OS (*P* = 0.211) for del19 patients.

In our study, patients with squamous cell carcinoma had significantly higher rates of elevated SCCA than those with adenocarcinoma. Only in the squamous cell carcinoma group did serum SCCA level serve as an independent predictive and prognostic marker, consistent with a previous report [15]. Within these patients, SCCA levels did not vary depending on EGFR mutation status. Due to the low frequency of elevated SCCA in EGFR-mutant adenocarcinoma patients, we could not analyze its prognostic value.

A previous study found that the prognostic value of NSE for stage I NSCLC is limited [31], while another reported that NSE was correlated with lactate dehydrogenase, tumor diameter, and disease extent as well as a predictor of survival [12]. Similarly, Yu et al. demonstrated that high levels of preoperative serum NSE correlated with worse survival in NSCLC patients [32]. We found that NSE was significantly higher in squamous cell carcinoma than in adenocarcinoma cases, although was unable to predict recurrence or OS. NSE levels were higher in adenocarcinoma cases with EGFR-mutations and only within L858R subgroup did NSE level have marginal predictive and prognostic significance. The discrepancy between our study and others could be attributed to different sample size of patients, or the inconstancy of co-variables introduced in the proportional hazards model in previous studies.

Our retrospective study here has some limitations, as confounding factors cannot be reduced as much as in prospective, randomized studies. It should be noted that treatment after recurrence clearly had an impact on overall survival, especially the use of EGFR-TKI for patients harboring EGFR mutations. In our cohort most recurrent patients received cisplatin-based doublet chemotherapy, while only 54 received EGFR-TKI therapy. However, recurrent patients treated with EGFR-TKI were evenly distributed into EGFR del19 and L858R subgroups. This may minimize the impact of the therapies administered after recurrence. Secondly, only EGFR del19 and L858R were examined in this study; therefore, uncommon EGFR mutations might have been miscategorized during analysis. Third, it is unclear whether alterations in serum tumor marker levels actually represent the intratumor heterogeneous components in each patient, although Tanaka et al. found that EGFR-TKI efficacy in EGFR-mutated patients depended on the initial Cyfra21-1 level and concluded this represented a squamous-rich component in NSCLC [8]. Despite of these limitations, the cases analyzed in our study were in accordance with the uniform inclusion and exclusion criteria, which strengthens our confidence in the results and provides potentially useful information for clinical practice.

In conclusion, Cyfra21-1 is a predictive and prognostic marker in resectable adenocarcinoma patients harboring EGFR mutations, and a prognostic factor in EGFR del19 or L858R group. However, CEA was an independent predictive and prognostic factor only for EGFR wild-type adenocarcinoma patients and EGFR L858R adenocarcinoma patients. A prospective clinical trial is necessary to validate our present findings.

## MATERIALS AND METHODS

### Patients

1016 NSCLC patients treated by curative-intent complete resection between January 2008 and October 2012 at the Tianjin Medical University Cancer Institute & Hospital were investigated retrospectively. The study was approved by the Institutional Review Board of Tianjin Medical University Cancer Institute & Hospital and informed consent was obtained from all patients. Exclusion criteria were: (1) locally advanced (stage IIIB), metastasized (stage IV), or postsurgically relapsed NSCLC; (2) preoperative chemotherapy or radiotherapy; (3) history of second primary cancer diagnosed within 5 years; and (4) patients who died within 30 days after resection. Preoperative evaluation included physical examination, blood chemistry analysis, measurement of serum tumor markers, bronchofiberscopy, chest radiograph, computed tomography (CT), brain magnetic resonance imaging (MRI) or CT and bone scintigraphy. All patients underwent a wedge resection, lobectomy or pneumonectomy and systematic dissection of hilar and mediastinal lymph nodes for resection of the primary lesion. Complete resection was defined as complete removal of all tumors with negative margin proven by histopathological examination. Diagnosis of malignant disease was confirmed pathologically and classified according to World Health Organization histological classification (3rd edition) and staged according to the TNM classification of the Union for International Cancer Control (7th edition). Primary adjuvant treatment after surgery was chemotherapy or radiotherapy, either alone or in combination. Platinum-based adjuvant chemotherapy was routine for stage IB-IIIA NSCLC. Patients with multistation mediastinal N2 disease received sequential radiotherapy following completion of adjuvant chemotherapy. Follow-up information was collected directly from the outpatient clinic records or from family contact. Patients were evaluated every 3 months by chest CT scans for the first 2 years after surgery and annually thereafter. The date of recurrence, treatment for recurrence, date of death or last visit, and cause of death were recorded. Patient characteristics are shown in Table 1.

### Measurement of serum CEA, NSE, SCCA and Cyfra21-1 levels and EGFR mutations

Serum concentrations of CEA, NSE, SCCA and Cyfra21-1 were measured within 2 weeks before surgery by electrochemiluminescence immunoassay on Roche Analytics E170 Immunology Analyzer (Roche Diagnostics, China). Based on manufacturer recommendation, the following cut-offs for serum marker levels were used: CEA 5.0 ng/ml, NSE 15.2 ng/ml, SCCA 1.5 ng/ml, and Cyfra21-1 3.3 ng/ml. EGFR mutations (del19 and L858R) were identified by real-time PCR or DNA sequencing as previously described [33]. Other EGFR mutations were not tested.

### Statistical analysis

Continuous variables were described using mean ± SD or median and range. Comparison of average value between groups was detected with rank sum test. Categorical variables were compared by chi-square test or Fisher's exact test. DFS was defined as the interval between lung resection and local recurrence and/or occurrence of distant metastases. Overall survival time was calculated as the interval between surgery and death or last clinical evaluation. Survival was estimated by the Kaplan-Meier method and compared using the Log-rank test for univariate analysis. The hazard ratio (HR) and the 95% confidence intervals (CI) were estimated using the Cox proportional hazard model, and the multivariate Cox model was developed using stepwise regression (backward selection) to adjust for potential confounding factors. *P* values of less than 0.05 were considered statistically significant. SPSS (version 18.0; SPSS, Inc., Chicago, IL) was used for all analyses.

## References

[1] Pao W, Chmielecki J (2010). Rational, biologically based treatment of EGFR-mutant non-small-cell lung cancer. Nat Rev Cancer.

[2] Russo A, Franchina T, Ricciardi GR, Picone A, Ferraro G, Zanghi M, Toscano G, Giordano A, Adamo V (2015). A decade of EGFR inhibition in EGFR-mutated non small cell lung cancer (NSCLC): Old successes and future perspectives. Oncotarget.

[3] Mok TS, Wu YL, Thongprasert S, Yang CH, Chu DT, Saijo N, Sunpaweravong P, Han B, Margono B, Ichinose Y, Nishiwaki Y, Ohe Y, Yang JJ (2009). Gefitinib or carboplatin-paclitaxel in pulmonary adenocarcinoma. N Engl J Med.

[4] Yang JC, Wu YL, Schuler M, Sebastian M, Popat S, Yamamoto N, Zhou C, Hu CP, O'Byrne K, Feng J, Lu S, Huang Y, Geater SL (2015). Afatinib versus cisplatin-based chemotherapy for EGFR mutation-positive lung adenocarcinoma (LUX-Lung 3 and LUX-Lung 6): analysis of overall survival data from two randomised, phase 3 trials. Lancet Oncol.

[5] Zhang Y, Sheng J, Kang S, Fang W, Yan Y, Hu Z, Hong S, Wu X, Qin T, Liang W, Zhang L (2014). Patients with exon 19 deletion were associated with longer progression-free survival compared to those with L858R mutation after first-line EGFR-TKIs for advanced non-small cell lung cancer: a meta-analysis. PloS One.

[6] Kuan FC, Kuo LT, Chen MC, Yang CT, Shi CS, Teng D, Lee KD (2015). Overall survival benefits of first-line EGFR tyrosine kinase inhibitors in EGFR-mutated non-small-cell lung cancers: a systematic review and meta-analysis. Br J Cancer.

[7] Zhu JQ, Zhong WZ, Zhang GC, Li R, Zhang XC, Guo AL, Zhang YF, An SJ, Mok TS, Wu YL (2008). Better survival with EGFR exon 19 than exon 21 mutations in gefitinib-treated non-small cell lung cancer patients is due to differential inhibition of downstream signals. Cancer lett.

[8] Tanaka K, Hata A, Kaji R, Fujita S, Otoshi T, Fujimoto D, Kawamura T, Tamai K, Takeshita J, Matsumoto T, Monden K, Nagata K, Otsuka K (2013). Cytokeratin 19 fragment predicts the efficacy of epidermal growth factor receptor-tyrosine kinase inhibitor in non-small-cell lung cancer harboring EGFR mutation. J Thorac Oncol.

[9] Chiu CH, Chou TY, Chiang CL, Tsai CM (2014). Should EGFR mutations be tested in advanced lung squamous cell carcinomas to guide frontline treatment?. Cancer Chemother Pharmacol.

[10] Grunnet M, Sorensen JB (2012). Carcinoembryonic antigen (CEA) as tumor marker in lung cancer. Lung Cancer.

[11] Wang XB, Li J, Han Y (2014). Prognostic significance of preoperative serum carcinoembryonic antigen in non-small cell lung cancer: a meta-analysis. Tumour biol.

[12] Ferrigno D, Buccheri G, Giordano C (2003). Neuron-specific enolase is an effective tumour marker in non-small cell lung cancer (NSCLC). Lung Cancer.

[13] Pujol JL, Molinier O, Ebert W, Daures JP, Barlesi F, Buccheri G, Paesmans M, Quoix E, Moro-Sibilot D, Szturmowicz M, Brechot JM, Muley T, Grenier J (2004). CYFRA 21–1 is a prognostic determinant in non-small-cell lung cancer: results of a meta-analysis in 2063 patients. Br J Cancer.

[14] Niklinski J, Burzykowski T, Niklinska W, Laudanski J, Chyczewski L, Rapellino M, Furman M (1998). Preoperative CYFRA 21–1 level as a prognostic indicator in resected nonsmall cell lung cancer. Eur Respir J.

[15] Vassilakopoulos T, Troupis T, Sotiropoulou C, Zacharatos P, Katsaounou P, Parthenis D, Noussia O, Troupis G, Papiris S, Kittas C, Roussos C, Zakynthinos S, Gorgoulis V (2001). Diagnostic and prognostic significance of squamous cell carcinoma antigen in non-small cell lung cancer. Lung Cancer.

[16] Yang ZM, Ding XP, Pen L, Mei L, Liu T (2014). Analysis of CEA expression and EGFR mutation status in non-small cell lung cancers. Asian Pac J Cancer Prev.

[17] Pan JB, Hou YH, Zhang GJ (2013). Correlation between EGFR mutations and serum tumor markers in lung adenocarcinoma patients. Asian Pac J Cancer Prev.

[18] Jin B, Dong Y, Wang HM, Huang JS, Han BH (2014). Correlation between serum CEA levels and EGFR mutations in Chinese nonsmokers with lung adenocarcinoma. Acta Pharmacol Sin.

[19] Pan JB, Hou YH, Zhang GJ (2014). Correlation between efficacy of the EGFR tyrosine kinase inhibitor and serum tumor markers in lung adenocarcinoma patients. Clin Lab.

[20] Jung M, Kim SH, Hong S, Kang YA, Kim SK, Chang J, Rha SY, Kim JH, Kim DJ, Cho BC (2012). Prognostic and predictive value of carcinoembryonic antigen and cytokeratin-19 fragments levels in advanced non-small cell lung cancer patients treated with gefitinib or erlotinib. Yonsei Med J.

[21] Facchinetti F, Aldigeri R, Aloe R, Bortesi B, Ardizzoni A, Tiseo M (2015). CEA serum level as early predictive marker of outcome during EGFR-TKI therapy in advanced NSCLC patients. Tumour Biol.

[22] Fiala O, Pesek M, Finek J, Benesova L, Minarik M, Bortlicek Z, Topolcan O (2014). Predictive role of CEA and CYFRA 21–1 in patients with advanced-stage NSCLC treated with erlotinib. Anticancer Res.

[23] Wieskopf B, Demangeat C, Purohit A, Stenger R, Gries P, Kreisman H, Quoix E (1995). Cyfra 21–1 as a biologic marker of non-small cell lung cancer. Evaluation of sensitivity, specificity, and prognostic role. Chest.

[24] Hotta K, Segawa Y, Takigawa N, Kishino D, Saeki H, Nakata M, Mandai K, Eguchi K (2000). Evaluation of the relationship between serum carcinoembryonic antigen level and treatment outcome in surgically resected clinical-stage I patients with non-small-cell lung cancer. Anticancer Res.

[25] Barlesi F, Gimenez C, Torre JP, Doddoli C, Mancini J, Greillier L, Roux F, Kleisbauer JP (2004). Prognostic value of combination of Cyfra 21–1, CEA and NSE in patients with advanced non-small cell lung cancer. Respir Med.

[26] Okada M, Nishio W, Sakamoto T, Uchino K, Yuki T, Nakagawa A, Tsubota N (2004). Prognostic significance of perioperative serum carcinoembryonic antigen in non-small cell lung cancer: analysis of 1, 000 consecutive resections for clinical stage I disease. Ann Thorac Surg.

[27] Fang S, Wang Z, Guo J, Liu J, Li C, Liu L, Shi H, Liu L, Li H, Xie C, Zhang X, Sun W, Li M (2014). Correlation between EGFR mutation status and response to first-line platinum-based chemotherapy in patients with advanced non-small cell lung cancer. OncoTargets Ther.

[28] Toyooka S, Takano T, Kosaka T, Hotta K, Matsuo K, Ichihara S, Fujiwara Y, Soh J, Otani H, Kiura K, Aoe K, Yatabe Y, Ohe Y (2008). Epidermal growth factor receptor mutation, but not sex and smoking, is independently associated with favorable prognosis of gefitinib-treated patients with lung adenocarcinoma. Cancer Sci.

[29] Shoji F, Yoshino I, Yano T, Kometani T, Ohba T, Kouso H, Takenaka T, Miura N, Okazaki H, Maehara Y (2007). Serum carcinoembryonic antigen level is associated with epidermal growth factor receptor mutations in recurrent lung adenocarcinomas. Cancer.

[30] Zhang Y, Jin B, Shao M, Dong Y, Lou Y, Huang A, Han B (2014). Monitoring of carcinoembryonic antigen levels is predictive of EGFR mutations and efficacy of EGFR-TKI in patients with lung adenocarcinoma. Tumour Biol.

[31] Ma S, Shen L, Qian N, Chen K (2011). The prognostic values of CA125, CA19. 9, NSE, AND SCC for stage I NSCLC are limited. Cancer Biomark.

[32] Yu D, Du K, Liu T, Chen G (2013). Prognostic value of tumor markers, NSE, CA125 and SCC, in operable NSCLC Patients. Int J Mol Sci.

[33] Jiang R, Jin Z, Liu Z, Sun L, Wang L, Li K (2011). Correlation of activated STAT3 expression with clinicopathologic features in lung adenocarcinoma and squamous cell carcinoma. Mol Diagn Ther.

